# Implementation of a complex intervention to improve participation in older people with joint contractures living in nursing homes: a process evaluation of a cluster-randomised pilot trial

**DOI:** 10.1186/s12877-020-01655-z

**Published:** 2020-08-05

**Authors:** Hanna Klingshirn, Martin Müller, Katrin Beutner, Julian Hirt, Ralf Strobl, Eva Grill, Gabriele Meyer, Susanne Saal

**Affiliations:** 1 Institute for Medical Information Processing, Biometry and Epidemiology, Ludwig-Maximilians-Universität München, Marchioninistr 17, 81377 Munich, Germany; 2 Faculty of Applied Health and Social Sciences, Rosenheim Technical University of Applied Sciences, Hochschulstraße 1, 83024 Rosenheim, Germany; 3 Institute for Health and Nursing Sciences, Medical Faculty, Martin Luther University Halle-Wittenberg, Magdeburger Straße 8, 06112 Halle (Saale), Germany; 4 German Centre for Vertigo and Balance Disorders, Ludwig-Maximilians-Universität München, Marchioninistr 15, 81377 Munich, Germany

**Keywords:** Joint contractures, Nursing homes, Participation, Complex intervention, Cluster-randomised controlled trials, Pilot study, Implementation strategy, Process evaluation

## Abstract

**Background:**

Joint contractures in frail older people are associated with serious restrictions in participation. We developed the Participation Enabling CAre in Nursing (PECAN) intervention, a complex intervention to enable nurses to promote participation in nursing home residents with joint contractures. The aim of this study was to examine the feasibility of the implementation strategy and to identify enablers and barriers for a successful implementation.

**Methods:**

The implementation of PECAN was investigated in a 6-month pilot cluster-randomised controlled trial (c-RCT). As a key component of the implementation strategy, nominated nurses were trained as facilitators in a one-day workshop and supported by peer-mentoring (visit, telephone counselling). A mixed-methods approach was conducted in conjunction with the pilot trial and guided by a framework for process evaluations of c-RCTs. Data were collected using standardised questionnaires (nursing staff), documentation forms, problem-centred qualitative interviews (facilitators, therapists, social workers, relatives, peer-mentors), and a group discussion (facilitators). A set of predefined criteria on the nursing home level was examined. Quantitative data were analysed using descriptive statistics. Qualitative data were analysed using directed content analysis.

**Results:**

Seven nursing homes (*n* = 4 intervention groups, *n* = 3 control groups) in two regions of Germany took part in the study. Facilitators responded well to the qualification measures (workshop participation: 14/14; workshop rating: “good”; peer-mentor visit participation: 10/14). The usage of peer-mentoring via telephone varied (one to seven contacts per nursing home). Our implementation strategy was not successful in connection with supplying the intervention to all the nurses. The clear commitment of the entire nursing home and the respect for the expertise of different healthcare professionals were emphasised as enablers, whereas a lack of impact on organisational conditions and routines and a lack of time and staff competence were mentioned as barriers.

**Conclusion:**

The PECAN intervention was delivered as planned to the facilitators but was unable to produce comprehensive changes in the nursing homes and subsequently for the residents. Strategies to systematically include the management and the nursing team from the beginning are needed to support the facilitators during implementation in the main trial.

**Trial registration:**

German clinical trials register, DRKS00010037. Registered 12 February 2016.

## Background

Joint contractures are characterised as restrictions of the physiological movement of any joint because of deformity, disuse or pain [1]. Older people living in nursing homes are particularly often affected by joint contractures due to the association with several health conditions, immobility and age. Prevalence varies between 20 and 75% in studies involving nursing home residents as a result of different definitions and hardly comparable populations [1–5]. Irrespective of the underlying aetiology, living with a joint contracture can be severely disabling for the affected individual. An impairment of the upper extremities may reduce the capacity to perform daily activities like dressing or eating, while an impairment of the lower extremities may reduce the ability to walk independently and increase the risk of bed confinement [6, 7]. Recent research, using the International Classification of Functioning, Disability and Health (ICF) [8] as a framework, indicates that joint contractures are associated with numerous limitations of functioning such as mobility, self-care, sensory function and pain, domestic life and community, social and civic life [9]. Limitations in activities (i.e., “the execution of a task or action”) and restrictions in participation (i.e., “the involvement in a life situation”) are the most relevant problems for the affected individuals [9–13]. Moreover, interviews with affected individuals in geriatric care revealed that immobility does not necessarily lead to restrictions in participation, rather the restrictions are induced by environmental and personal factors [9].

Existing interventions do not consider the complexity of the phenomenon of joint contracture. Despite the multiple causes of joint contractures, currently used interventions for prevention and treatment are mainly single interventions [14–16], which are not effective in multimorbid, older people and do not consider the outcomes that are most relevant to residents like activities and participation [16]. Due to diverse treatment priorities, a wide range of healthcare professionals are involved in the care of individuals with joint contractures, for example nurses, physical and occupational therapists and physicians. The involvement of informal caregivers is also crucial [12]. A successful intervention for nursing home residents with joint contractures has to consider the interaction between joint contractures, the individuals’ daily life and the influence of environmental and personal factors, and should also address all healthcare professionals involved in the treatment of the affected individuals [17]. Therefore, the intervention must by its very nature be complex.

In the JointConImprove project [18] we carefully developed such a complex intervention called the “Participation Enabling CAre in Nursing” (PECAN) intervention [17]. The development followed the UK Medical Research Council (MRC) framework [19] and systematically integrated existing evidence [16], best practice models, the expertise of healthcare professionals [12], and the perspective of the affected individuals [9, 11]. The development of the PECAN intervention is reported in detail elsewhere [17]. For newly developed interventions, the UK MRC framework recommends a pilot testing phase [19]. Consequently, the second part of the JointConImprove project [18] was to test the PECAN intervention in a pilot cluster-randomised controlled trial (c-RCT) accompanied by a detailed process evaluation.

Particularly in a pilot trial, the key function of a process evaluation is to understand the feasibility and acceptability of the implementation strategy and the proposed evaluation design [20]. Since the examination of the proposed evaluation design and the feasibility of the implementation strategy raise different sets of research questions, we decided to report the results separately. The results of the PECAN pilot trial with focus on the feasibility of the proposed study design is reported elsewhere [21].

This paper aims to examine the feasibility and acceptability of the PECAN implementation strategy and to identify enablers and barriers for a successful implementation.

## Methods

### The PECAN pilot trial

The full pilot trial details are reported elsewhere [21]. In summary, the PECAN pilot trial was planned as a multi-centre pragmatic trial with a two-armed, parallel group design. Ethical approval was obtained from the responsible ethics committees. Residents were included if they were aged 65 years or older and affected by at least one joint contracture diagnosed by a physician, therapist or nurse. Residents suffering from the terminal stage of a disease were excluded. Seven nursing homes (i.e. the clusters) with a total of 129 residents were recruited from a convenience sample in two regions of Germany. Prior to the start of the study, all the residents (and/or the legal guardians) were asked for a written informed consent by the research team. Structured face-to-face interviews by blinded assessors were used to collect residents’ data at baseline, then after 3 and 6 months. The primary outcome was defined as the residents’ participation and measured with the PaArticular Scales [22]. The secondary outcomes were defined as residents’ activities, instrumental activities of daily living, health-related quality of life, as well as falls and fall-related consequences to ensure the safety of the intervention. After baseline assessment, four nursing homes with 64 participating residents were randomised to the intervention group (PECAN) and three nursing homes with 65 residents were randomised to the control group (optimised standard care i.e., standard care including an information session addressing general aspects of care for residents with joint contractures).

### Study design of the process evaluation

A mixed-methods process evaluation was employed with data collection in conjunction with the PECAN pilot c-RCT. As recommended for process evaluation studies, we applied quantitative methods to assess whether the key processes of the implementation followed the study protocol and qualitative methods to determine enablers and barriers during the implementation [20]. Quantitative and qualitative data were given equal consideration, as they complement each other in a deeper interpretation of the findings [23].

We applied the MRC guidance for the evaluation of complex interventions by Moore et al. [20] along with the framework proposed by Grant et al. for the design and reporting of process evaluations for c-RCTs [24]. Grant et al. differentiate in their framework between processes involving clusters, processes involving individuals and their interaction with the context in which the trial is embedded [24]. Since the PECAN intervention is delivered first to the nursing homes and not directly to the residents, this process evaluation focuses on processes involving the nursing homes (i.e. the clusters) in order to improve the implementation strategy for the main trial. We used the Standards for Reporting Implementation Studies (StaRI) Statement [25] for reporting our implementation and the Template for Intervention Description and Replication (TIDieR) checklist [26] for reporting our intervention.

### The PECAN intervention

Based on the biopsychosocial model of the ICF [8], the core idea of the PECAN intervention is to facilitate a participation-oriented understanding of care in nursing homes, to allow improved analysis of the residents’ situation and to guide the nursing home staff in their decision-making. The individually tailored PECAN intervention focuses on the dynamic interaction between an individual’s health condition and existing personal and environmental factors that can act as facilitators or barriers for performing activities and for participation [8, 17].

#### Process of change

The mechanisms of the expected changes in the nurses’ professional behaviour to improve the residents’ participation are based on the principles of the Theory of Planned Behaviour (TPB) [27], which is a proven theory to predict or explain the behaviour of healthcare professionals [28, 29]. Intermediate intervention goals to change the behaviour of the nursing home staff are presented in the logic model of the PECAN intervention in Additional file 1, Figure A1.

#### Implementation strategy

The key aspect of the PECAN implementation strategy is the facilitation approach [30]. Facilitation is the active part of the implementation, carried out by trained facilitators, who guide individuals or organisations through a challenging change process [30, 31]. As change agents, facilitators are responsible for guiding the implementation and for offering education and counselling to their colleagues. The implementation of PECAN proceeds in multiple steps: In the first step, the intervention is introduced to skilled nurses, who are trained as facilitators. The research team guided the delivery of the intervention throughout all the nursing homes. In the second step, the facilitators are responsible for the integration of the PECAN intervention into the daily practice by involving, counselling or educating the nursing team, physicians, therapists, social workers and relatives. During this process the facilitators were supported by experienced peer-mentors, who were members of the research team [17].

**Fig. 1:**
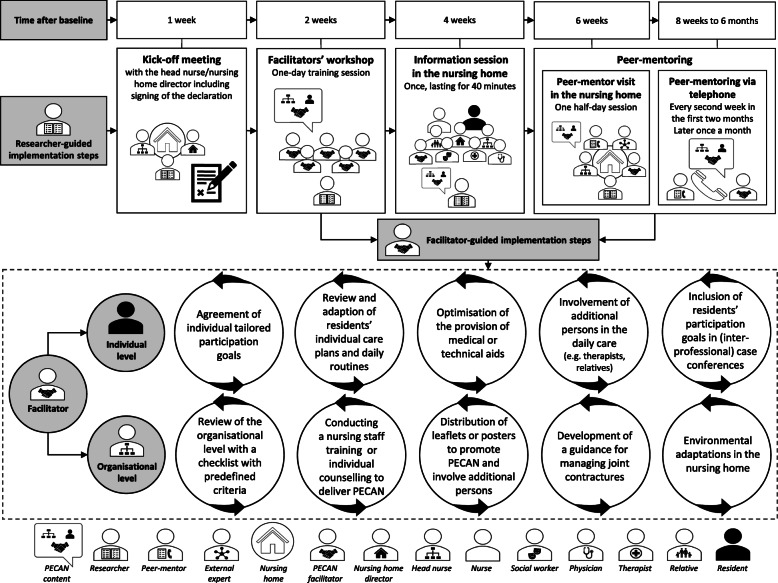
Overview of the PECAN implementation strategy

An overview of the PECAN implementation strategy is presented in Fig. 1.

#### Researcher-guided implementation steps

##### Kick-off meeting with the head nurse/nursing home director

In the *kick-off meeting*, the intervention was introduced to the head nurse and/or the director of the nursing home, who signed a declaration ensuring their commitment.

##### Facilitators’ workshop

The key component of the implementation was a one-day *facilitators’ workshop* to prepare nominated skilled nurses who have received a degree for their role as facilitator following at least 3 years of formal vocational education. Based on predefined qualification criteria (e.g., formal vocational education) the facilitators were selected by the head nurse. During the workshop, the intervention was explained, including comprehensive information on the phenomenon of joint contractures, a training session on how to implement residents’ participation goals in individual care planning using the biopsychosocial model of the ICF, and a training session on peer counselling methods [32] to involve all team members in the implementation process and to improve interprofessional collaborations.

##### Information session

A single *information session* lasting 40 min was held by a member of the research team in each nursing home for the residents, relatives, nursing staff and other interested healthcare professionals (regardless of their participation in the study). In the intervention group the aim of the session was to introduce the PECAN intervention, the facilitators and their tasks, and to provide ideas about how everybody could support the implementation. In the control group the aim of the session was to inform about risks and consequences of joint contractures, to introduce the study and provide contact to the research team.

##### Peer-mentoring

The facilitators were supported via a mentoring approach, where they received counselling by a trained mentor (a nurse in the research team). Starting with the *peer-mentor visit* in each nursing home, a mentor and an external peer expert gave the facilitators counselling and support in evaluating and adapting implementation measures tailored for their institution. Using structured assessment tools, the facilitators reviewed the residents’ individual care plans and the organisational procedures (in collaboration with the head nurse) in order to identify barriers and enablers for the residents’ participation. Based on this review, the facilitators developed a tailored action plan for the implementation of PECAN in their nursing home. During the implementation process, the peer-mentor supported the facilitators in transforming their plans into action. Changes at the organisational level were realised in collaboration with the *peer-mentor*, the head nurse and the facilitator. Following the visit, *peer-mentoring* was conducted via phone calls from their mentors every second week in the first 2 months and later once a month. The peer-mentors were free to offer fixed and regular counselling appointments or to provide counselling only if required. The peer-mentors at both study centres shared their experiences in regular telephone meetings and discussed with a third member of the research team any problems that arose during peer-mentoring.

##### Supportive materials

Posters and other written materials were provided to inform and remind nursing home staff and residents. Outpatient therapists, physicians and relatives were addressed by leaflets with customised information about the intervention and contact details of the facilitators.

#### Facilitator-guided implementation steps

To achieve the intervention goals, an individually tailored approach is used including both the individual (i.e., resident) and the organisational (i.e., nursing home) level.

##### Individual level

The residents’ activities and participation were addressed by defining individual participation goals and their care plans and daily routines were accordingly reviewed and adapted. Measures to meet the participation goals on the individual level contained, for example, the use of a biographical approach to identify the residents’ potential motivation for activities and participation, the inclusion of residents’ participation goals in (interprofessional) case conferences, the optimisation of the provision of medical or technical aids and the involvement of additional persons in the daily care by peer counselling and by using project leaflets for external therapists, physicians or relatives when it is necessary to reach residents’ participation goals.

##### Organisational level

The review and change process to integrate the perspective of the ICF was guided by using a checklist with predefined criteria. In consultation with the head nurse the facilitators promoted changes on the organisational level to disseminate the PECAN principles. This included nursing team training sessions, individual counselling, the distribution of leaflets and posters, the de-novo-development of a guidance for managing joint contractures according the core aspects of the PECAN intervention or the adaptation of an existing guidance, environmental adaptations in the nursing home, as well as the redistribution of tasks involving the nursing home management, the nursing team and the interprofessional team (i.e., social workers, physicians and therapists) [17].

### Standard care – the context

In Germany, nursing homes are financed by the German statutory long-term care insurance and additional payment from the residents. On a legal basis, 50% of the nursing staff had to be skilled nurses with at least 3 years of vocational training. Nursing home residents are frequently affected by age-related disorders and multimorbidity. Social activities are usually planned by in-house social care assistants and social workers. Physicians and therapists typically do home visits to the nursing homes. Medical and technical aids as well as physical therapy, occupational therapy and speech and language therapy need to be prescribed by a physician and are financed by the German statutory long-term care insurance with a co-payment from the residents.

### Study population of the process evaluation

The study population of this process evaluation included all persons who were closely engaged in the implementation of PECAN and provided the perspective of

- the facilitators, responsible for the implementation of PECAN,
- the nurses, who were introduced to the intervention by the facilitators,
- additional persons, who were closely engaged in the care of residents with joint contractures, i.e., therapists, social workers and relatives,
- and the research team, especially the trained peer-mentors, who were responsible for support of the facilitators during implementation.

The nursing team included skilled nurses, nursing assistants, nursing students and social care assistants, since they represent the nursing team in each nursing home ward. Therapists were physical or occupational therapists employed by the nursing home or by an outpatient practice. Social workers were employed by the nursing home and were responsible for supporting residents in independent living and social participation, e.g., organisation and coordination of individual and group offers. Relatives were defined as a family member or a legal guardian of a participating resident and were randomly selected by the research team based on the participants’ list of the residents. The residents had already been involved in the feasibility testing of the study procedures and were asked to participate in structured face-to-face interviews. We decided to exclude residents from the process evaluation of the interventions’ implementation to keep the burden of questioning as low as possible for the residents in this pilot trial [21].

### Data collection

**Fig. 2:**
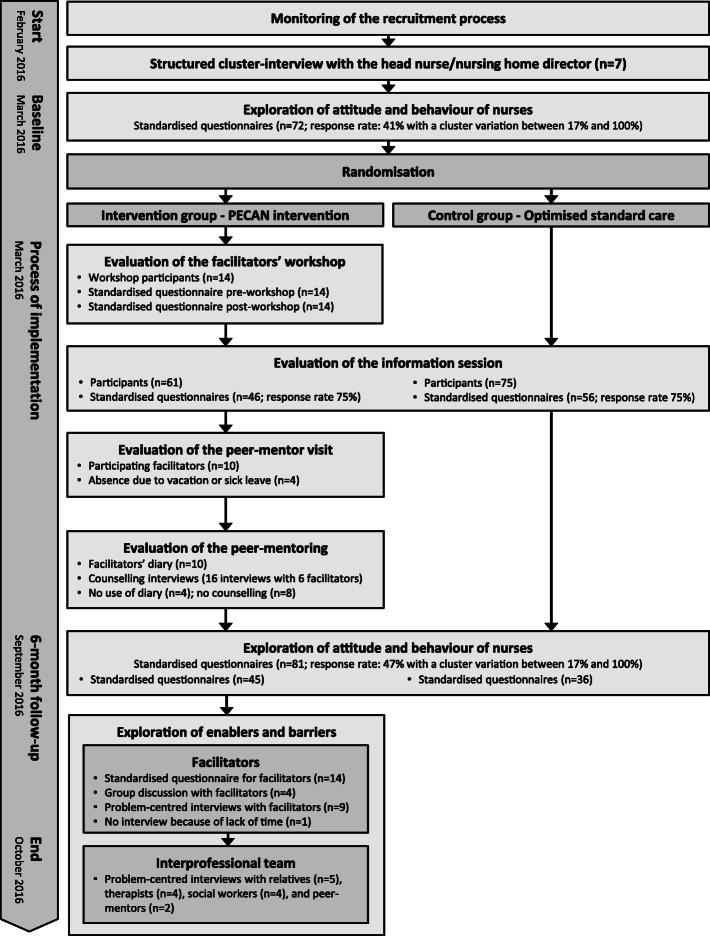
Flow of process evaluation

Data were collected prior to, during and post- intervention to illustrate changes over time [20]. Figure 2 displays the flow of the process evaluation. During data collection we focussed on the components “delivery to clusters” (i.e., process where the research team delivers intervention content to the nursing home), “response of clusters” (i.e., process where the nursing home adopts intervention content into daily nursing care), and “the context” (i.e., anything external to the intervention) which might be an interacting component [24]. An overview of the components and data collection methods of the process evaluation for the PECAN intervention adapted from Grant et al. [24] is presented in Table 1.

**Table 1:** Components and methods of the process evaluation for the PECAN intervention adapted from Grant et al. (2013) [24]

Domain	Research question	Research methods and measures	Participants	Stage of study
**Delivery to clusters**	What intervention is actually delivered to each nursing home?	Evaluation of the facilitators workshop using documentation forms	Research team	During and after each implementation component
		Evaluation of the information session using documentation forms	Research team	
	Were the components of the implementation introduced as planned?	Evaluation of the peer-mentor-visit using documentation forms	Research team	
		Evaluation of the peer-mentoring using documentation forms	Research team	
**Response of clusters**	How is the intervention adopted by the nursing homes?	Feedback on implementation components and process using standardised questionnaires, documentation forms, and facilitators’ diary	Facilitators	During implementation and post-intervention
	Are there any differences between the nursing homes?		Participants in the information session	
			Research team	
	Are there any changes in daily nursing routine?	Survey using standardised questionnaire on experiences and perceived changes in attitude and behaviour	Nursing staff	At baseline and after 6 months
	What are the enablers and barriers for a successful implementation?	Problem-centred interviews and group discussion to ask about experiences during implementation	Facilitators	Post-intervention
			Therapists, social workers and relatives	
			Peer-mentors	
**Context**	In what context is the intervention implemented?	Description of the wider context based on literature on national nursing home standards	Literature search	Before baseline
		Collection of important structural characteristics using structured cluster-interviews	Head nurse	At baseline
	How do contextual factors influence the implementation process?	Problem-centred group interviews and group discussion to ask about the influence of context-specific factors during implementation	Facilitators	Post-intervention

#### Characteristics of nursing homes – the context

Characteristics of the included nursing homes were collected at baseline via structured interviews with the head nurse or the director of the nursing home.

#### Process of implementation

The *facilitators’ workshop* and the *information session* were evaluated by their participants with standardised questionnaires to assess content-related (e.g., relevance for professional development, practical relevance) and educational aspects (e.g., structure, comprehensibility, quality of training materials). As overall feedback, the participants rated the events on a scale ranging from 1 = “excellent” to 6 = “inadequate”. The predefined qualification for the role of facilitators was reviewed in detail as part of the survey (e.g., formal vocational education). The participants in the *information session* were asked whether they were nurses, relatives, residents, or members of other groups.

Standardised documentation forms were used by the research team to review the implementation process according to protocol. We assessed the attendance in the *information session* (number and group affiliation of participants), the fidelity of the *peer-mentor visit* (number of participants, procedure according to protocol), the fidelity of the counselling interviews during *peer-mentoring by telephone* (content, number of interviews per facilitator, interview duration), and amount and type of *supportive materials* used (e.g., leaflets, poster). To gain insight into the content of the intervention at the nursing home level, the facilitators’ activities during the implementation process were summarised in the facilitators’ diary.

#### Attitude and behaviour of nurses

A standardised questionnaire was used for a survey on the nurses’ professional attitude and behaviour in order to reach the target 20% subgroup of nursing staff in a short time. The questionnaires were distributed by the head nurse in the intervention group and control group at baseline and at the 6-month follow-up (convenience sample). Participants were randomly selected based on their presence (staff roster) during the data collection period. Nurses were asked to rate six statements about the care of residents with joint contractures to verify to what extent the PECAN intervention is associated with a professional change in behaviour. Three additional statements regarding the reach of the intervention were rated exclusively in the intervention group at the 6-month follow-up. All statements were rated on a 5-point Likert scale (1 = “strongly agree” to 5 = “strongly disagree”; with “don’t know” as a sixth option).

#### Enablers and barriers of implementation

After the intervention period a detailed insight into the experiences of all stakeholders was needed. Therefore, all the facilitators were invited to join a group discussion in their respective study centre. Facilitators who could not join in were asked to participate in a problem-centred interview. Relatives, therapists, social workers, and the trained peer-mentors were also invited to take part in problem-centred interviews.

Both the problem-centred interviews and the group discussion followed semi-structured interview guides. To identify key enablers and barriers of a successful implementation, questions were asked regarding how the intervention was delivered, who was reached, how every single implementation component was experienced, and which factors were influencing the implementation.

The group discussion was moderated by one researcher (HK) and a study assistant at the study centre. The problem-centred interviews were conducted by single researchers (HK, JH, KB) at the participants’ workplace or at home via telephone. All the interviewers were trained by the research team in methods of leading group discussions [33] and problem-centred interviews [34]. The interviews and the group discussion were audio recorded. Field notes were taken and summarised in a post-script.

### Data analysis

Quantitative data were analysed by descriptive statistics using SAS Version 9.4 [35].

Qualitative data from the problem-centred interviews and group discussions were analysed using a mixed deductive-inductive approach based on the structured approach of directed content analysis [36]. Audio records of the group discussion and the interviews were “abridged transcribed” [33] with priority given to relevant contents by members of the research team (HK, JH, KB). Meaningful examples of quotations from the participants were transcribed verbatim. For quality assurance reasons, the participants were offered the opportunity to review and modify the transcripts.

Two researchers (HK, KB) developed a coding guideline based on one transcript from each group of participants. To finalise the coding guideline, categories were cross-compared and discussed until a consensus was reached [37]. The final coding guideline was reviewed by two senior researchers (MM, SuS). Any data that could not be categorised with the initial coding guideline were assigned to a new sub-category. Where reasonable, the description of the categories was based on the categories of the ICF, which was the conceptual model used to design the intervention [8, 38]. The data analysis was supported by MAXQDA Version 12 [39]. The results were classified into enablers and barriers.

Qualitative data from documentation forms or minutes and field notes were classified inductively into categories, based on the content of the given answers.

## Results

### Characteristics of nursing homes – the context

Seven nursing homes (*n* = 4 intervention groups, *n* = 3 control groups) in two regions of Germany took part in the study. The number of long-term care beds varied between 40 and 171 across the nursing homes. Within the nursing homes, the number of wards ranged from two to six wards, the ratio of nursing staff to residents for skilled nurses was 0.19 in total (cluster-variation between 0.16 and 0.28), and the prevalence of joint contractures varied between 19 and 96%. All nursing homes conducted interprofessional case conferences (five on a regular basis, two on an occasional basis). The services in the local environment varied, but four of the seven nursing homes were in walking distance to parks, stores, churches, and coffee bars. Five of the seven nursing homes have an environment that promotes physical activity with therapeutic gardens or walking circuits. The characteristics of the nursing homes are presented in Table 2.

**Table 2:** Characteristics of nursing homes (adapted from Saal et al. 2019) [21]

	Intervention group				Control group			Total
	Cluster 1	Cluster 2	Cluster 3	Cluster 4	Cluster 5	Cluster 6	Cluster 7	
Study participants	9	20	11	24	24	23	18	129
Participants levels of care dependency^a^								
None	0	0	1	0	0	0	0	1
Low	0	0	0	0	0	0	2	2
Considerable	5	14	3	1	10	1	7	41
Severe	4	6	6	8	11	9	7	51
Most severe	0	0	1	15	3	13	2	34
Ownership ^b^	*private*	*private*	*church-owned*	*church-owned*	*non-profit*	*non-profit*	*private*	
Long-term care beds	40	107	171	165	48	128	115	774
Nursing home wards	3	4	4	6	2	4	6	29
Residents per ward	13	27	43	28	24	32	18	27
Prevalence of joint contractures ^c^	0.40	0.96	0.19	0.21	0.50	0.31	0.60	0.28
Ratio of nursing staff to residents								
Skilled nurses and assistants	0.49	0.30	0.35	0.38	0.32	0.34	0.30	0.35
Skilled nurses	0.28	0.16	0.19	0.20	0.17	0.16	0.16	0.19
Interprofessional case conferences ^d^	*regularly*	*occasionally*	*regularly*	*regularly*	*regularly*	*occasionally*	*regularly*	
Local environment ^e^								
Park areas	*yes*	*yes*	*yes*	*yes*	*no*	*yes*	*yes*	
Stores (e.g. supermarket, drugstore)	*no*	*yes*	*yes*	*yes*	*no*	*yes*	*yes*	
Churches	*no*	*no*	*yes*	*yes*	*no*	*yes*	*yes*	
Coffee bars	*no*	*yes*	*yes*	*yes*	*no*	*yes*	*yes*	
Environment promoting physical activity^f^	*no*	*no*	*yes*	*yes*	*yes*	*yes*	*yes*	
Degree of urbanisation ^g^	*rural*	*urban*	*urban*	*suburban*	*suburban*	*urban*	*suburban*	

^a^Levels of care dependency as assessed by expert raters from the medical service of the German statutory health insurance system

^b^Categorisation of ownership = non-profit, private, state-owned, or church-owned

^c^Prevalence estimated by the head nurse

^d^Categorisation of the conduction of interprofessional case conferences = regularly, occasionally, or never

^e^Defined as close to the nursing home within walking distance for the residents

^f^Defined as movement-promoting architectural features in or outside the nursing home e.g. therapeutic garden, barrier-free walking circuits, handrails, wheelchair accessibility

^g^Defined by degree of urbanisation acc. to the statistical office of the European office (Eurostat) = urban, suburban, or rural

### Process of implementation

Results on the degree of implementation of the PECAN intervention are presented in Table 3. Results on enablers and barriers of the PECAN implementation strategy from the problem-centred interviews are summarised in Table 4.

**Table 3:** Implementation of the PECAN intervention

	Cluster 1	Cluster 2	Cluster 3	Cluster 4
**Kick-off meeting**				
Meeting conducted according to protocol	✓	✓	✓	✓
Declaration signed	✓	✓	✓	✓
**Facilitators’ workshop**				
Agenda and content according to protocol	✓	✓	✓	✓
Number of trained facilitators	2/2	2/2	4/4	6/6
Qualification for the role as facilitator	2/2	2/2	4/4	6/6
**Information session**				
Session conducted according to protocol	✓	✓	✓	✓
Number of participants per session				
Nursing staff	0	2	11	11
Residents	4	3	3	0
Relatives	1	1	0	2
Others	0	1	1	1
Missing	0	3	1	1
Total	5	10	16	15
**Peer-mentoring**				
**Peer-mentor visit**				
Agenda and content according to protocol	✓	✓	✓	✓
Number of facilitators participating	2/2	2/2	2/4	4/6
Participation of the head nurse	✓	✓	✓	✓
Support by an external peer-expert	✓	✓	–	✓
**Peer-mentoring via telephone**				
Number of counselling interviews	6	7	1	2
Number of facilitators counselled	2/2	2/2	1/4	1/6
Interview duration in minutes, mean (range)	85 (105–30)	31 (75–10)	10 (10–10)	13 (10–15)
**Supportive materials**				
Project leaflets given to the nursing homes	10	10	30	30
Specific leaflets for relatives, therapists, physicians given to the nursing homes	35	40	21	21
Posters to promote physical activity given to the nursing homes	3	3	4	6
Set of material for nursing team training	–	–	4	7
Article for nursing home journal	–	–	1	–
**Facilitators’ diary**				
Response of the diary	2/2	1/2	3/4	4/6
Monthly working time per facilitator in hours, mean (range)	20 (20–20)	5 (5–5)	19 (17–20)	5 (1–10)

**Table 4:** Enablers and barriers of the PECAN implementation strategy

Categories	Enablers	Barriers
**Overall strategy**	• Stepwise training of facilitators (i.e., facilitators’ workshop, peer-mentor visit, peer-mentoring via telephone) (F)	• Lack of systematic involvement of all the different stakeholders (i.e., management, social workers, relatives, and therapists) (F, R, T, SW)
	• Clear defined PECAN content (F)	• Available time period too short to complete implementation (F)
	• Personal contact initiated by the management or the facilitators to provide the different stakeholders with information on PECAN (T, F)	• Difficulties in the implementation for residents with severe physical and cognitive impairment (F)
**Facilitators’ workshop**	• Practical elements (e.g., training on the use of technical and medical aids) (M)	• Unbalanced ratio between theory and practice (i.e., more active participation during workshop required) (F, RT)
**Information session**	• Use of plain language when addressing the different participant groups (RT)	• Lack of systematic involvement of the nursing staff (e.g., no presentation within the nursing team) (F)
	• Diverse groups of participants could be reached and informed about PECAN in one session (F, SW)	• Invitation to the session (i.e., poster at the entrance area) did not reached all potential participants (F, T, R, SW, RT)
**Peer-mentoring**	• The peer-mentor visit was highlighted as a useful introduction to the implementation of PECAN (F)	• Facilitators were usually not directly available via e-mail or telephone (e.g., appointments via the head nurse were necessary) (F, PM)
	• Continuous availability of the peer-mentors via telephone (F)	
	• Standardised procedure of peer-mentoring via telephone (F, PM) - Routines for communication and regular appointments (F, PM) - Specific objectives based on the last counselling (PM)	
**Supportive materials**	• Supportive materials tailored for the target population (F, T, SW) - Training folder for facilitators (F) - Posters for the nursing wards (T, SW, F) - Materials for nursing team training (F) - Specific leaflets for relatives, therapists and physicians (F) - Article regarding PECAN published in nursing home journal (SW)	• Lack of supportive materials with a simple and practical design (F, R)
		• Lack of supportive materials to guide the implementation (e.g., no standardised documentation forms, no overview of potential intervention measures) (F)
		• Leaflets should have more focus on personal tasks (R)
		• Supportive materials did not reach the targeted population (R, T, SW) - Posters or other reminders in the nursing wards were not noticed (R) - Leaflets were not handed out (R, T, SW)

*Abbreviations*: *RT* research team, *F* facilitators, *R* relatives, *T* therapists, *SW* social workers, *PM* peer-mentors

**Data base:** Statements from the research team based on documentation forms (2 protocols for the facilitators’ workshop, 2 protocols for the information session); statements from the facilitators based on problem-centred interviews (9 participants) and one group discussion (4 participants); statements from relatives (5 participants), therapists (4 participants) and social workers (4 participants) based on problem-centred interviews; statements from the peer-mentors based on problem-centred interviews (2 participants)

Out of the 57 persons invited to the problem-centred interviews, 28 persons took part, 13 facilitators (13/14), five relatives (5/24), four therapists (4/13), four social workers (4/4), and the two peer-mentors (2/2). The response was particularly high among internal stakeholders (facilitators and social workers), while only a few external stakeholders (therapists and relatives) responded to the invitation distributed by the head nurse.

The head nurse or nursing home director of each nursing home signed the declaration to ensure their commitment to improve residents’ participation and to support the implementation of PECAN. In the *facilitators’ workshop*, 14 nurses from two study regions and four nursing homes (2 to 6 nurses per nursing home) were trained as facilitators as planned. All the facilitators fulfilled the predefined qualification criteria and had at least 1 year of professional experience (range: 1 to 11 years). In addition, seven facilitators had at least one advanced vocational training in nursing (gerontological psychiatry nursing *n* = 2; palliative care nursing *n* = 3; case management *n* = 1; nursing management *n* = 4; clinical instructor *n* = 3). Whereas in clusters 2, 3 and 4 all the facilitators were engaged in daily nursing care on their ward, one of the facilitators in cluster 1 was the deputy nursing home director.

The topics of the workshop were mainly rated as highly relevant for practice (high *n* = 10; partly *n* = 4; low *n* = 0). After the workshop, 13 out of 14 facilitators felt competent to be active in the adaptation of care plans. Further information about the self-assessed preparedness for the role as facilitator is presented in Additional file 1, Table A1. Overall, the quality of the *facilitators workshop* was rated with 1.7 points (SD 0.45; range: 1 to 2 points), indicating a good acceptance of the workshop.

Findings from the problem-centred interviews present a more detailed picture: The theoretical part of the workshop, in which the existing evidence on the development and prevention of joint contractures was conveyed, was found to be not really instructive, on the other hand the practical elements of the workshop were judged as particularly relevant for daily care.

Facilitator (F3, C2) about the theoretical part of the workshop:

*I had thought that maybe I would learn something new, [...] but that was not the case.*

Facilitator (F1, C1) about the practical part of the workshop:

*What I liked very much was that someone from the medical supply store was there. I thought it was really good that he had said something too.*

The *information session* was conducted in all clusters according to protocol. A total of 136 participants from seven nursing homes (intervention group *n* = 61; control group *n* = 75) attended the *information session*; 102 participants (range: 5 to 16 participants per nursing home) completed a questionnaire (response rate: 75%). Out of these 102 attendants, the proportion of nursing staff, residents, and relatives varied widely between the clusters (Table 3). Overall, the quality of the *information session* was rated with 1.9 points (SD 0.76; range: 1 to 4 points), indicating a good acceptance of the session. The statement by a relative points out why in some nursing homes external participants rarely receive information about the events taking place in the nursing home.

Relative (R2, C3) about the poster with the announcement for the *information session*:

*[ … ] there's a bulletin board a little further back in the hall, but there are a thousand notes. I don’t really take notice of it.*

From the perspective of the facilitators, the session should have reached more nurses.

Facilitator (F13, C4) about the participation of nurses in the information session:

*There [should have been] many more employees, perhaps this should have taken place at a different time.*

Regardless of their participation in the information session, it became apparent that the content of the session was not detailed enough for the nurses. In the problem-centred interviews, some facilitators therefore suggested a short training session for all the nurses.

Facilitator (F12, C4) about the training of nursing staff:

*[...] the head nurse could already decide that [...] I can indeed explain what we have discussed - what the purpose of the intervention is - but to conduct a compulsory training session is a different matter [...]. For one or two hours.*

*Peer-mentoring* (*peer-mentor visit, peer-mentoring by telephone, supportive material)* was offered to all the nursing homes. Due to sick leave and vacation occurrences, four out of 14 facilitators were unable to participate during the *peer-mentor visit*. Overall, the *peer-mentor visit* was highlighted by the facilitators as a useful introduction to implementing PECAN.

Facilitator (F11, C4) about the *peer-mentor visit*:

*It was especially interesting [...] at that time we introduced our residents, you [the researchers] also got to know our residents. That was really, really great.*

During the visit the facilitators used a structured assessment tool to review organisational procedures and to develop tailored action plans to implement PECAN into their nursing home. In addition, case conferences were conducted at each visit, and individual care plans were developed for two residents to improve their participation. Support was given by the peer-mentor (all clusters) and an external peer expert (cluster 1, 2 and 4).

The action plans were realised with support of the peer-mentor during the following weeks. In total, 16 counselling interviews were conducted, with strong variation between clusters (between one and seven counselling interviews per nursing home), and facilitators (6 of 14 facilitators received counselling). The mean interview duration was 48 min with a range from 10 to 85 min (Table 3).

The main counselling topics were individual residents’ care, therapeutic care, use of technical and medical aids, interprofessional collaboration, collaboration with relatives, organisational needs, and implementation activities. The number of counselling interviews is associated with the different methods of both peer-mentors (the first peer-mentor was responsible for cluster 1 and 2; the second peer-mentor was responsible for cluster 3 and 4). Whereas the first peer-mentor imparted a mandatory procedure with fixed appointments right from the start and structured counselling based on specific objectives, the second peer-mentor imparted an optional approach and invited the facilitators to initiate contact themselves whenever counselling was needed. The standardised procedure of counselling with routines for communication and regular appointments was emphasised by both facilitators and peer-mentors as being supportive.

Facilitator (F1, C1) about the peer-mentor:

*The mentoring by one of the researchers who continually inquired or provided incentives and motivations … it has always been quite good that there was someone else to ask.*

Peer-mentor (P1):

*What worked well was my commitment to my contacts. [...] I had defined clear communication paths and tools right from the start.*

All the nursing homes used the offered *supportive materials*, especially leaflets offering information on the PECAN intervention and the study procedure for relatives, therapists and physicians, as well as posters for promoting physical activity. Additional materials were used in accordance with the individual needs of the nursing homes (Table 3). The problem-centred interviews highlighted the impact to provide supplementary materials to support the implementation.

Facilitators (F13, C4):

*Yes, your information material was an advantage, we could hang up the posters. Well, someone always took a look at it.*

Facilitator (F8, C3):

*A special supplement for the documentation is missing.*

The facilitators adopted various measures to implement the PECAN intervention in their nursing homes. The analysis of the facilitators’ diaries (*n* = 10 diaries returned out of 14) revealed that the following measures were conducted in all nursing homes: Adaptation of nursing records and care planning, development of an institution-specific guidance for managing joint contractures, inclusion of residents’ participation goals in case conferences with the nursing staff and the interprofessional team, counselling of colleagues and relatives, discussions with superiors, social workers, therapists and physicians, review of technical and medical aids, and environmental adaptations in the residents’ area and the nursing home. The documentation from the peer counselling and the problem-centred interviews provided better information about what was happening in the nursing homes.

For example on the individual level, in cluster 2 the review of medical aids resulted in the necessity to replace a walker with a more suitable one. Another resident in cluster 2, has been using a wheelchair since moving into the nursing home, although the nurses believed he would be still able to walk short distances. Therapists and nurses agreed to encourage the resident to become more involved in transfers and use a walker in his room.

At the organisational level, cluster 1 organised an interprofessional in-house workshop to optimise the provision of medical or technical aids. The workshop was conducted 6 weeks after the visit in cooperation with the medical supply store. In addition to the nursing staff and the advisor from the medical supply store, external therapists and the *peer-mentor* took part to support the training. In cluster 4, the facilitators introduced the PECAN intervention to their nursing team, using the posters and material sets for nursing team training in team meetings, and integrated the intervention in the daily handovers and case conferences.

#### Attitude and behaviour of nurses

The response of nursing staff to the PECAN intervention after 6 months is presented in Table 5.

**Table 5:** Response of the nursing staff to the PECAN intervention after 6 months

Do you agree with the following statements?	Cluster 1 (*n* = 10)		Cluster 2 (*n* = 12)		Cluster 3 (*n* = 6)		Cluster 4 (*n* = 17)		Total (*n* = 45)	
	n	(%)	n	(%)	n	(%)	n	(%)	n	(%)
**I feel well informed about PECAN.**										
Agree	10	(100)	1	(8)	4	(66)	13	(77)	28	(62)
Neutral	0		0		2	(33)	2	(12)	4	(9)
Disagree	0		11	(92)	0		2	(12)	13	(29)
**Supportive materials (e.g., posters, handouts, leaflets) on PECAN were provided comprehensively.**										
Agree	10	(100)	1	(8)	3	(50)	13	(77)	27	(60)
Neutral	0		3	(25)	0		2	(12)	5	(11)
Disagree	0		8	(66)	3	(50)	2	(12)	13	(29)
**The facilitators provided counselling whenever it was needed.**										
Agree	10	(100)	3	(25)	3	(50)	12	(71)	28	(62)
Neutral	0		1	(8)	0		2	(12)	3	(7)
Disagree	0		7	(58)	3	(50)	2	(12)	12	(27)
Missing	0		1	(8)	0		1	(6)	2	(4)
**Overall, are you satisfied with the implementation of PECAN in your nursing home?**										
Extremely / very satisfied	10	(100)	1	(8)	4	(67)	12	(71)	27	(60)
Moderately satisfied	0		2	(17)	1	(17)	5	(29)	8	(18)
Not at all / slightly satisfied	0		5	(42)	1	(17)	0		6	(13)
Don’t know	0		4	(33)	0		0		4	(9)

All in all, some of the nurses disagreed (“strongly disagree” and “disagree”) that they felt well informed about PECAN (13/45, 29%), that comprehensive *supportive materials* were provided (13/45, 29%) and that the facilitators provided counselling whenever it was needed (12/45, 27%). After 6 months, the overall satisfaction of the nurses (“extremely” and “very satisfied”) with the implementation of PECAN varied strongly between the nursing homes (cluster-variation between 8 and 100%). Particularly in cluster 2, the majority of the nurses felt poorly informed about the PECAN intervention (11/12, 92%) and were dissatisfied with the implementation (5/8, 42%). The interview with the peer-mentor revealed that especially in cluster 2 the facilitators had no support from the nursing home director, which made it impossible for them to realise their role and to involve the nursing staff in initiating changes. In contrast, a facilitator from cluster 3 describes his role as being only supportive to counselling colleagues and instigating changes.

Peer-Mentor (P1) about cluster 2:

*[...] it was not at all possible [ … ] to realise the role as facilitator, i.e. the facilitator had the task after the training [...] of passing on the [contents of the intervention] to the colleagues. This was not successful at all in the larger institution. The support of the nursing home director was lacking.*

Facilitator (F8, C3):

*In the role [as facilitator] I was able to assert myself better. I could say "Come, let's go to the resident and then you show me how you do it".*

To identify changes in daily routines due to the PECAN intervention, the nurses in the intervention group as well as in the control group were asked to rate statements towards organisational aspects that contribute to the residents’ participation (Additional file 1; Table A2). For example, in the intervention group, two thirds of the nurses (30/45, 67%) agreed (“strongly agree” and “agree”) with the statement “We often discuss how to improve the care of residents with joint contractures to enable them to participate in social life in the best possible way” at the 6-month follow-up, while less than half of the nurses agreed to this statement at baseline (22/51, 43%) or at the 6-month follow-up in the control group (17/36, 47%).

### Enablers and barriers at the nursing home level

Enablers and barriers of implementation at the nursing home level are summarised in Table 6.

**Table 6:** Enablers and barriers of implementation at the nursing home level

Categories	Enablers	Barriers
**Personal factors**	• Social relationships (F) - Respect and social support of facilitators by the nursing team (F)	• Social relationships (F) - Therapists perceive PECAN as an interference in their responsibilities (F) - Conflicting opinions and challenges within the interprofessional team regarding the care of residents with joint contractures (F, T)
		• Motives and motivation (F, SW, R) - Differing priorities of management and nursing team (F) - Poor motivation or little interest of the different stakeholders, i.e., nurses (F), physicians (F), therapists (F), social workers (SW) or residents (R) - Lack of interprofessional attitude among physicians (F) - Uncertainty and fear among relatives (e.g., additional costs, overburdening) (F)
**Organisational factors**	• Clear commitment of the entire nursing home (F) - Active leadership to support changes (e.g., regularly occurring agreements and exchange, adoption of organisational tasks, approved time slots for meetings, provision of technical and medical aids) (F) - Open-mindedness to changes in the nursing team (e.g., review of residents’ care plans, implementation of measures to support participation, initiation of case conferences) (F) - Clear responsibilities within the interprofessional team (e.g., in collaboration with social workers, therapists and physicians) (F)	• Lack of impact on organisational conditions and routines (F, SW, T, R) - Unclear and unspecified responsibilities (F, SW) - Lack of interprofessional collaboration (e.g., little exchange, strict separation of working areas) (F, SW, T, R) - No established culture of contact and exchange between relatives and nursing staff (R) - No interprofessional case conferences (SW, T)
	• Respect for the expertise of different healthcare professionals and relatives (F, SW, T, R) - Respect for involved healthcare professionals (F, SW, T, R) - Recognition of various expertise and resources (T, SW, R)	• Lack of time and staff competences (F, R, T) - Staff shortage and high workload for nurses (F, R, T) and therapists (F, T) - No time slots for unscheduled tasks (F) - Skills shortage in the nursing staff (F, R, T) - Language barriers of the nursing staff (R)

*Abbreviations*: *F* facilitators, *R* relatives, *T* therapists, *SW* social workers

**Data base:** Statements from the facilitators based on problem-centred interviews (9 participants) and one group discussion (4 participants). Statements from relatives (5 participants), therapists (4 participants) and social workers (4 participants) based on problem-centred interviews

Implementation at the nursing home level is influenced by the personal characteristics of the different stakeholders and by the organisational and structural conditions of the nursing homes. Moreover, there are differences between the included clusters and between the perceptions of the stakeholders. For example, the facilitators experienced the social relationship, which includes the open-mindedness of staff towards the PECAN intervention, in different ways.

Facilitator (F1, C1):

*It’s hard... to really convince these die-hard nurses to actively participate, to implement, to think, to observe. That is difficult [...], and they must really want it.*

Facilitator (F12, C4):

*Now something is happening here and I felt it was positive that we were practically involved. Half [of the nursing staff] could also have said “Oh, I don't feel like it” [...] or “I'm not interested in that here”.*

As a fundamental precondition for a successful implementation, the clear commitment of the entire nursing home is required. This covers an active leadership in supporting the changes, open-mindedness to the changes, and clear responsibilities. These quotes from two facilitators illustrate how commitment can be experienced and, in contrast, how implementation stagnates if there is no commitment by the nursing home.

Facilitator (F9, C4):

*We were always exempted from work for the meetings. For discussions, we got extra time. [...] It was a very, very close collaboration.*

Facilitator (F6, C3):

*I missed the togetherness [...]. I had talked to the head nurse after our workshop [...], but I had the impression ‘yes, that's nice you were here’ [...]. I missed the commitment and the interest.*

Moreover, a successful implementation is motivated by respecting the expertise of the different stakeholders, as emphasized in the following quote.

Facilitator (F1, C1):

*And I also have to say, the whole solidarity between us all, nurses, physical therapists, physicians, occupational therapists, this is now a really good collaboration, it works, you complement each other, you get tips.*

A lack of impact on organisational conditions and routines was identified as a major barrier for the implementation. This includes unclear responsibilities and a lack of interprofessional collaboration which was impeded by the strict separation of working areas and the lack of an established culture of change. The subsequent quote by a therapist addresses the problem of the documentation.

Therapist (T3, C2):

*[...] we have a documentation obligation as therapists. However, the documentation is run via our practice and not the nursing home. Well, I don't have to explain what I did in the nursing home, but that's normal.*

A barrier that was reported as important across all clusters and from different stakeholders was a lack of time and staff competence, as illustrated by the subsequent quotes:

Social worker (S2, C2):

*Well, it’s not like I’m closed off to communication, for example. But very often it’s a time problem. That you don’t take enough time to share information or to communicate.*

Facilitator (F6, C3):

*The major problem is of course the staff shortage, this is still known in many nursing homes [...] the time of course [...] whether management or staff, everyone has to do his work, is a bit stressed [...]*

## Discussion

This process evaluation describes the implementation of the PECAN intervention for the first time and emphasises enablers and barriers for a successful implementation. The implementation process was coordinated by the facilitators and included tailored measures to integrate the perspective of the ICF into daily nursing care. Although the intervention was delivered to the facilitators by the research team as planned, it was not passed on properly to the nurses, healthcare professionals, relatives and, subsequently, to the residents.

During the implementation process, differences between the nursing homes became apparent. While in cluster 1 all the nursing staff surveyed were satisfied with the implementation of the intervention, the nurses in cluster 2 were not satisfied with the implementation. Cluster 1 is a comparably small nursing home in which the support of the management was assured, since one of the two facilitators held the position of the deputy nursing home director. Moreover, the facilitators in cluster 1 invested a lot of time in the implementation and also made intensive use of *peer-mentoring*. In contrast, cluster 2 had limited support from the nursing home management due to personnel changes, which eventually led to termination of the implementation at the nursing home level.

In our study, we identified the clear commitment of the entire nursing home and the respect for the expertise of different healthcare professionals as main enablers for a successful implementation. The most important barriers were a lack of impact on organisational conditions and routines, and a lack of time and staff competence. Therefore, our study reveals strengths and difficulties of the PECAN implementation strategy and suggests that specific optimisations are required.

The applied facilitation approach is a proven strategy for implementing interventions in nursing homes and for supporting changes in the daily nursing routine [40–43]. A successful implementation of knowledge into practice depends on the quality and type of the evidence, existing specific nursing home characteristics and the modalities of facilitation [30]. Our results confirmed the stepwise training of facilitators as an appropriate implementation strategy to empower facilitators. Nevertheless, in our pilot study empowerment of a facilitator alone was not sufficient to change practice. Here, our results are in line with Aasmul et al., indicating that a successful implementation did not depend on the facilitator alone [40]. It turned out that the facilitators can only act successfully when they can rely on a working environment that is supportive to inducing changes. This includes the existing time resources and the colleagues’ open-mindedness for training and counselling. Considering the low participation of the nurses in the information session and their lack of information regarding the PECAN intervention, it is apparent that further implementation strategies are needed to ensure the reach of the intervention. As a complementary strategy we used critical review and adaption of existing guidance for managing joint contractures to initiate the change in practice. However, we failed to support the facilitator in translating the guidance into nursing home practice using the existing quality management infrastructure. A nursing staff training support by the nursing home quality management would have probably increased the acceptance of the PECAN intervention.

Another issue is that since 2008, social care assistants (qualified in 12 weeks) have been introduced in nursing homes to support nurses by managing and offering leisure activities for residents [44]. Accordingly, it might be reasonable to initiate joint care planning between nurses and social care assistants. This could be encouraged by inviting the head of the social care assistants to participate in the *facilitators’ workshop,* emphasising their common responsibility regarding activities for and participation of residents.

The *peer-mentor visit* was regarded as very beneficial, especially when the residents’ individual care plans were reviewed during case conferences, which are an established approach to improve the care of nursing home residents [45–47]. In our study, case conferences have also proven to be a useful strategy for the adoption of tailored intervention measures and for implementation processes in practice, particularly since the concept of the case conference had already been established in the nursing homes. The participation of the *peer-mentor* in a case conference would have been another useful measure to ensure a better implementation of the PECAN intervention. The use of routine communication mechanisms to ensure staff commitment is a proven measure to provide practice change [48]. Moreover, peer counselling methods [32] to advise and coach nurses during implementation were an important module of the *facilitators’ workshop*, which needs more practical training and discussion in an extra session. The *peer-mentoring* via *telephone* was mainly considered as an enabler for initiating changes, although the utilisation varied widely. Continuous support of facilitators via email, telephone or on-site visits is part of many interventions when working with facilitators [40, 41, 43]. The strong variation in the number of counselling interviews is associated with the different communication strategies of the two peer-mentors. In our study, a mandatory approach with fixed appointments right from the start, and a structured counselling based on specific objectives have proven themselves. Such standardised procedures with regular contacts during the implementation process have been reported as successful in other studies [40, 42]. Therefore, the training of peer-mentors should be extended, and the paths of communication should be further standardised. Our study found that *supportive materials* that are appropriate for everyday use and tailored for the targeted population were beneficial to imparting the intervention as simply and practically as possible. This is in line with Colón-Emeric et al. [49], who found that the balance between complexity and simplicity as well as the variety of delivery methods support the implementation success of behavioural change interventions in long-term care. Overall, the facilitators realised that a six-month study period was too short to complete the implementation, since some processes needed more time than scheduled in a pilot study.

Although there was a clear commitment of the entire nursing home, that was ensured by the adoption of a declaration to the PECAN intervention on the one hand, on the other hand there was a lack of staff commitment in organisation and practice change. During the implementation process, it became apparent in some clusters that the nursing management and the nursing staff had different priorities, that responsibilities were unclear, and that time slots for unscheduled tasks were not provided. While commitment is a precondition for change, change requires more effort than merely commitment. Several reasons might explain this paradox. First, despite detailed information on the PECAN implementation, nursing home managers seemed to underestimate the support needed by the facilitators. It is likely that more specific information about the responsibilities of the nursing home management might have increased the commitment. Second, staff turnover and sick leave limited the support by the nursing home management, especially in cluster 2. Therefore, the involvement of the quality management - not only as a deputy for the nursing home manager, but also as the existing infrastructure for inducing change – might have increased the practice change.

As in other studies [49, 50], we experienced that an active leadership component is important for initiating necessary organisational changes. In cluster 2, the nurses were dissatisfied with the implementation. This might have been caused by lack of support from the management, or because the vacancy of the head nurse was not filled over a longer period of time, which made the change process almost impossible. To increase the involvement of the head nurse, a structured approach with clearly defined responsibilities is needed. Moreover, an intensified relationship between the nursing home management and the collaborating partners is associated with the improvement of the residents’ health outcomes [51]. Our results suggest that a successful implementation needs mutual respect towards the expertise of different healthcare professionals, whereas a lack of impact on organisational conditions (i.e., unclear allocation of responsibilities, insufficient collaboration and interprofessional exchange) was identified as an important barrier. This finding is supported by D’Amour et al. [52], who identified two key elements for interprofessional collaboration: the creation of a common action that targets the complexity of client needs and the creation of a confident and respectful team culture that integrates the perspectives of all the professionals involved. Other studies indicate that a change of culture and staff practice is complex but feasible [50, 53]. The PECAN intervention tries to overcome existing barriers of interprofessional collaboration through the combination of measures on organisational and resident levels that are tailored to the needs of each nursing home and each individual resident.

In accordance with the results from a systematic review [53], we found that organisational factors such as a lack of time and staff competence or problems with maintaining routines were significant barriers for a successful implementation. The staffing situation was also highlighted as a context-specific barrier for the implementation. Staff shortages and excessive workloads are often described as barriers when providing an intervention [40, 54, 55]. The time pressure in nursing not only affects the nurses’ health-related quality of life but is also associated with a decreased quality of nursing care, and consequently, patient health outcomes [56]. Against this background, the PECAN intervention aims to qualify nurses in optimising organisational procedures and residents’ care without including additional time-consuming measures [17].

Overall, our study confirms the multi-step change mechanisms hypothesised with the underlying Theory of Planned Behaviour (TPB) [27]. The assumptions of the PECAN logic model, which indicated that the residents’ health status, time resources and the collaboration with different stakeholders are the influencing factors for a successful implementation, have been confirmed in this piloting phase [17].

### Strengths and limitations

This process evaluation has clear strengths. The PECAN intervention was developed according to the UK MRC framework [19], and is, with the background of the ICF [8], founded on a strong theoretical base in a field where evidence is sparse [17]. We used a multitude of proven implementation strategies in combination, which is in line with the expert recommendations for implementing change [57]. A feasibility testing stage is strongly recommended to avoid implementation or evaluation failure [20]. Although our intervention was developed with practitioners and nursing home experts [17], our piloting stage identified important optimisation needs for our implementation strategy. In addition, as a participation-orientated complex intervention, PECAN responds to a demand from a recent meta-analysis [58]. Herein, physical exercise interventions did not improve participation in older adults, and it was concluded that novel interventions are needed that should consider the individuals’ preferences as well as the physical, social and cultural environments. The PECAN intervention meets these requirements.

Moreover, we successfully adopted the framework proposed by Grant et al. [24] for c-RCTs and focused on processes involving clusters. The detailed description of the methods facilitates the replicability of the study processes. The included clusters varied in terms of size and staffing, which promotes the generalisability. As recommended for process evaluations [20], we integrated qualitative and quantitative methods to explain complex causal mechanisms.

Our study also has limitations. The response rate for some questionnaires was rather low. The challenge of conducting surveys with nursing staff is a well-known problem due to existing organisational, administrative and staff barriers [59]. Although we have tried to reduce the occurrence of socially desirable responses by ensuring a maximum of anonymity, it cannot be fully ruled out [60]. Therefore, the questionnaires’ results should be interpreted with caution. Qualitative interviews with the nursing staff and the residents in the main trial might be a more appropriate approach to get more in-depth information about the needs for support and perceptions of change in the nursing staff and residents. The recruitment of external stakeholders like therapists and relatives also proved difficult, since they were hardly included in the nursing home processes anyway.

Another limitation was the use of the facilitators’ diary which did not provide enough meaningful data. Although diaries or logs were often used to describe implementation processes [40, 61], in our study the use of a diary was insufficient to analyse the commitment of the facilitators to change culture and practice, as the response options were imprecise and the explanatory open-ended questions were not completed. We assume that in a setting where time resources are generally limited [62], methods with no additional documentation effort like a “diary interview” [63] would be more appropriate for the data collection in the main trial.

Finally, our study did not focus on processes involving the target population. In this pilot testing stage, our emphasis was on the implementation strategy, especially on how skilled nurses should be prepared to be facilitators and how facilitators should be supported during the implementation process. In a next step, it will be necessary to assess in more detail to what extent the intervention truly reaches the residents and what experiences the residents’ gain with the intervention.

## Conclusions

This process evaluation provides important insights into the implementation of a newly developed participation-orientated complex intervention in nursing homes. Pilot-testing the PECAN intervention identified essential optimisation needs for our implementation strategy. The intervention was delivered as planned to the facilitators but was insufficient to change the professional behaviour of the whole nursing staff in most clusters, and subsequently it failed to improve the residents’ participation. The main recommendations resulting from our study are likely to be applicable to any new developed nursing intervention. Our study found that a successful implementation does not depend on the facilitator alone. Focused strategies are needed to address further key stakeholders and to ensure the clear commitment of the entire nursing home during the whole implementation process. We recommend the use of existing structures of quality management and communication to ensure staff commitment, the enhancement of the peer-mentoring procedure with mandatory and regular contacts, and an approach to ensure an active leadership style from the head nurse to get an impact on organisational conditions and routines. In a next step, the optimised PECAN intervention will be investigated for its effectiveness and cost-effectiveness in a main trial accompanied by a revised process evaluation.

## Supplementary information

**Additional file 1: Figure A1.** Logic model of the Participation Enabling CAre in Nursing intervention; **Table A1.** Self-assessed preparedness for the role as facilitator after the workshop; **Table A2.** Nursing care of residents with joint contractures.

## Data Availability

The analysed datasets and the measurements used during the current study are available from the corresponding author on reasonable request.

## Abbreviations

c-RCT: cluster-randomised controlled trial
ICF: International Classification of Functioning, Disability and Health of the World Health Organization
MRC: Medical Research Council
PECAN: Participation Enabling CAre in Nursing
SD: Standard deviation
StaRI: Standards for Reporting Implementation Studies
TIDieR: Template for Intervention Description and Replication
TPB: Theory of Planned Behaviour
