# Supplementary material for: Implementation of a complex intervention to improve participation in older people with joint contractures living in nursing homes: a process evaluation of a cluster-randomised pilot trial
Source: BMC Geriatr. 2020 Aug 5;20:270. doi: 10.1186/s12877-020-01655-z (PMC7405353; doi:10.1186/s12877-020-01655-z)
Supplement: Supplementary file 1 — Additional file 1: Figure A1. Logic model of the Participation Enabling CAre in Nursing intervention; Table A1. Self-assessed preparedness for the role as facilitator after the workshop; Table A2. Nursing care of residents with joint contractures. [file 12877_2020_1655_MOESM1_ESM.pdf]

Implementation of a complex intervention to improve participation in older people with joint contractures living in nursing homes: A process evaluation of a cluster-randomised pilot trial

Table of Contents

Figure A1 Logic model of the Participation Enabling CAre in Nursing Intervention 2

Table A1 Self-assessed preparedness for the role as facilitator after the workshop 3

Table A2 Nursing care of residents with joint contractures 4

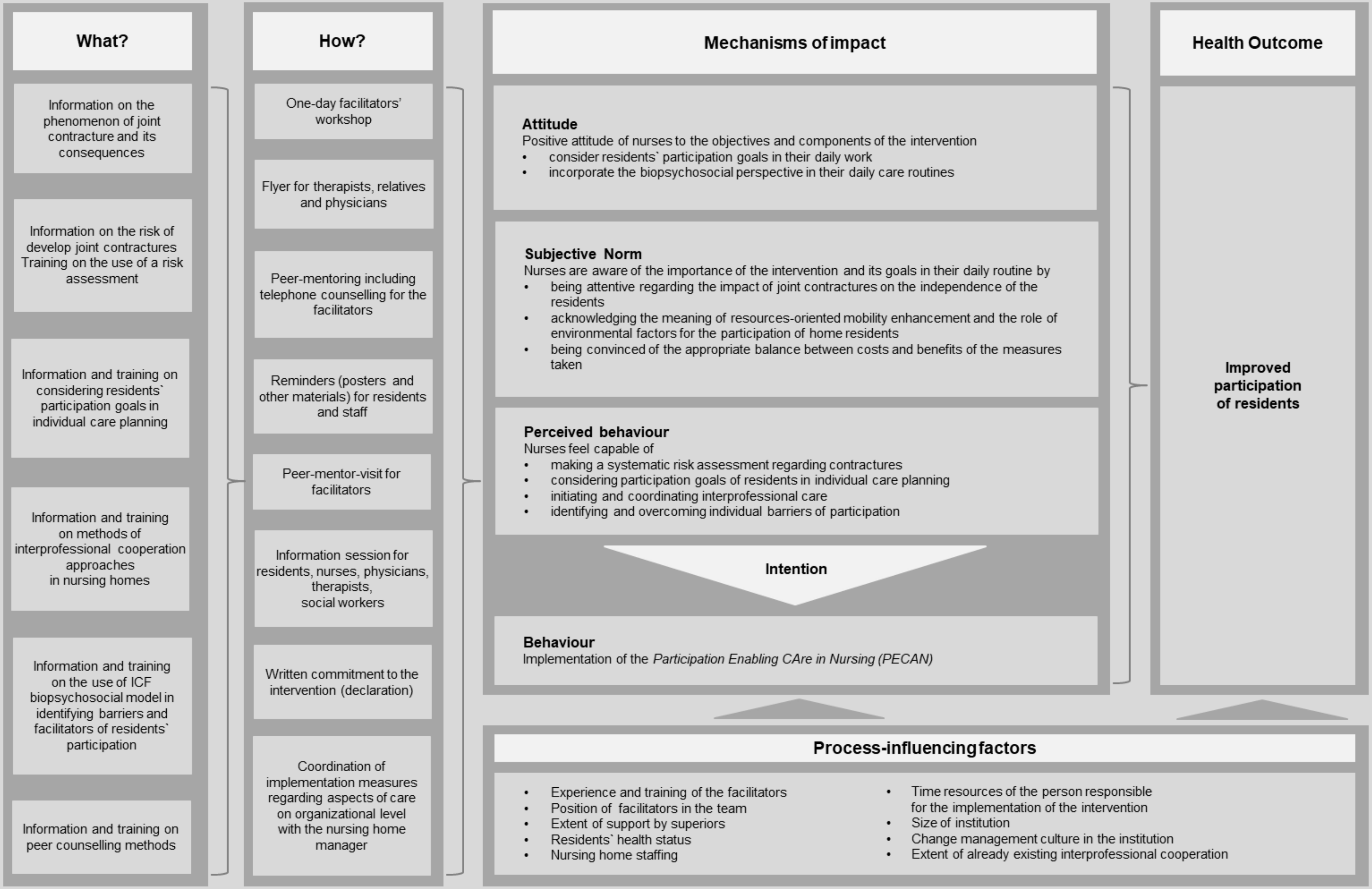

Figure A1 Logic model of the Participation Enabling CAre in Nursing Intervention adapted from Saal et al. (2018)

Saal S, Meyer G, Beutner K, Klingshirn H, Strobl R, Grill E et al. Development of a complex intervention to improve participation of nursing home residents with joint contractures: a mixed-method study. BMC Geriatr. 2018;18(1):61. doi:10.1186/s12877-018-0745-z.

Table A2 Self-assessed preparedness for the role as facilitator after the workshop

	n=14
Competence to use a biographical approach while planning measures.[1]	
Excellent / good	11
Less / not at all	3
Competence to encourage residents for activities.[1]	
Excellent / good	12
Less / not at all	2
Competence to enable the residents to participate without restrictions.[1]	
Excellent / good	12
Less / not at all	2
Competence to identify barriers and initiate changes in the nursing home.[1]	
Excellent / good	9
Less / not at all	4
Competence to be active in the adaptation of care plans.[1]	
Excellent / good	13
Less / not at all	1
Competence to review needs for technical and medical aids together with a therapist.[1]	
Excellent / good	11
Less / not at all	3
Confidence in the own performance of the role as facilitator[2]	
Excellent / good	7
Average	7
Less / not at all	0

[1]Based on a 4-Point-Likert-Scale (1=excellent, 2=good, 3=less, 4=not at all)
[2]Based on a 5-Point-Likert-Scale (1=excellent, 2=good, 3=average, 4=less, 5=not at all)

Table A2 Nursing care of residents with joint contractures

Do you agree with the following statements?	Baseline Intervention (n=51) n (%)	Baseline Control (n=21) n (%)	Baseline Total (n=72) n (%)	6-month follow-up Intervention (n=45) n (%)	6-month follow-up Control (n=36) n (%)	6-month follow-up Total (n=81) n (%)
**We often discuss how to improve the care of residents with joint contractures to enable them to participate in social life in the best possible way.**						
Agree	22 (43)	8 (38)	30 (41)	30 (67)	17 (47)	47 (58)
Neutral	13 (25)	6 (29)	19 (26)	10 (22)	12 (33)	22 (27)
Disagree	13 (26)	6 (29)	19 (26)	4 (9)	7 (19)	11 (14)
Don't know	3 (6)	1 (5)	4 (6)	1 (2)	0	1 (1)
**There are regular team meetings to discuss the care of residents with or at risk of joint contractures.**						
Agree	18 (36)	8 (38)	26 (36)	32 (71)	15 (41)	47 (59)
Neutral	11 (22)	3 (14)	14 (19)	3 (7)	12 (33)	15 (19)
Disagree	19 (37)	10 (48)	29 (41)	8 (18)	9 (25)	17 (21)
Don't know	3 (6)	0	3 (4)	2 (4)	0	2 (2)
**Individual biography and interests of residents with joint contractures are considered when planning activities and participation.**						
Agree	33 (65)	14 (67)	47 (65)	35 (77)	28 (78)	63 (78)
Neutral	6 (12)	4 (19)	10 (14)	7 (16)	4 (11)	11 (14)
Disagree	10 (20)	3 (15)	13 (18)	2 (4)	3 (8)	5 (6)
Don't know	2 (4)	0	2 (3)	1 (2)	1 (3)	2 (2)
**Independent action and mobility of residents with joint contractures is more important than the completion of a certain workload.**						
Agree	22 (43)	9 (43)	31 (43)	30 (67)	24 (67)	54 (67)
Neutral	8 (16)	6 (29)	14 (19)	8 (18)	6 (17)	14 (17)
Disagree	13 (26)	4 (19)	17 (23)	6 (13)	4 (11)	10 (12)
Don't know	7 (14)	2 (10)	9 (13)	1 (2)	1 (3)	2 (2)
Missing	1 (2)	0	1 (1)	0	1 (3)	1 (1)
**Residents with joint contractures are enabled to participate in individual daily activities like cooking, gardening or walking.**						
Agree	27 (53)	12 (57)	39 (54)	32 (71)	13 (36)	45 (56)
Neutral	10 (20)	3 (14)	13 (18)	6 (13)	10 (28)	16 (20)
Disagree	9 (18)	6 (28)	15 (21)	7 (15)	10 (27)	17 (21)
Don't know	4 (8)	0	4 (6)	0	3 (8)	3 (4)
Missing	1 (2)	0	1 (1)	0	0	0
**The collaboration with the residents' therapists works out well.**						
Agree	44 (86)	16 (76)	60 (84)	33 (73)	21 (58)	54 (67)
Neutral	5 (10)	2 (10)	7 (10)	8 (18)	8 (22)	16 (20)
Disagree	2 (4)	3 (15)	5 (7)	2 (4)	4 (12)	6 (8)
Don't know	0	0	0	2 (4)	3 (8)	5 (6)
